# Two years’ experience of implementing a comprehensive telemedical stroke network comprising in mainly rural region: the Transregional Network for Stroke Intervention with Telemedicine (TRANSIT-Stroke)

**DOI:** 10.1186/s12883-020-01676-6

**Published:** 2020-03-19

**Authors:** Katharina M. A. Gabriel, Steffi Jírů-Hillmann, Peter Kraft, Udo Selig, Viktoria Rücker, Johannes Mühler, Klaus Dötter, Matthias Keidel, Hassan Soda, Alexandra Rascher, Rolf Schneider, Mathias Pfau, Roy Hoffmann, Joachim Stenzel, Mohamed Benghebrid, Tobias Goebel, Sebastian Doerck, Daniela Kramer, Karl Georg Haeusler, Jens Volkmann, Peter U. Heuschmann, Felix Fluri

**Affiliations:** 1 Institute of Clinical Epidemiology and Biometry, University of Würzburg, Josef-Schneider-Straße 2/D7, 97080 Würzburg, Germany; 2 Neurology, Clinical Centre Main-Spessart, Lohr, Germany; 3 Neurology, Leopoldina Hospital Schweinfurt, Schweinfurt, Germany; 4 Neurology, Clinical Centre Rhön, Bad Neustadt/Saale, Germany; 5 Neurology, Clinical Centre Aschaffenburg-Alzenau, Aschaffenburg, Germany; 6 Neurology, Clinical Centre Würzburg Mitte, Standort Juliusspital, Würzburg, Germany; 7 Cardiology, Clinical Centre Helios Frankenwald, Kronach, Germany; 8 Internal Medicine, Clinical Centre Main, Ochsenfurt, Germany; 9 Neurology, Clinical Centre Helios Erlenbach, Erlenbach, Germany; 10 Internal Medicine, Clinical Centre Capio Franz von Prümmer, Bad Brückenau, Germany; 11 Neurology, University Hospital Würzburg, Würzburg, Germany; 12 Clinical Trial Center, University Hospital Würzburg, Würzburg, Germany; 13 Comprehensive Heart Failure Centre, University of Würzburg, Würzburg, Germany

## Abstract

**Background:**

Telemedicine improves the quality of acute stroke care in rural regions with limited access to specialized stroke care. We report the first 2 years’ experience of implementing a comprehensive telemedical stroke network comprising all levels of stroke care in a defined region.

**Methods:**

The TRANSIT-Stroke network covers a mainly rural region in north-western Bavaria (Germany). All hospitals providing acute stroke care in this region participate in TRANSIT-Stroke, including four hospitals with a supra-regional certified stroke unit (SU) care (level III), three of those providing teleconsultation to two hospitals with a regional certified SU (level II) and five hospitals without specialized SU care (level I). For a two-year-period (01/2015 to 12/2016), data of eight of these hospitals were available; 13 evidence-based quality indicators (QIs) related to processes during hospitalisation were evaluated quarterly and compared according to predefined target values between level-I- and level-II/III-hospitals.

**Results:**

Overall, 7881 patients were included (mean age 74.6 years ±12.8; 48.4% female). In level-II/III-hospitals adherence of all QIs to predefined targets was high ab initio. In level-I-hospitals, three patterns of QI-development were observed: a) high adherence ab initio (31%), mainly in secondary stroke prevention; b) improvement over time (44%), predominantly related to stroke specific diagnosis and in-hospital organization; c) no clear time trends (25%). Overall, 10 out of 13 QIs reached predefined target values of quality of care at the end of the observation period.

**Conclusion:**

The implementation of the comprehensive TRANSIT-Stroke network resulted in an improvement of quality of care in level-I-hospitals.

## Background

### Health care in rural areas

Access to specialized health care in rural areas is often restricted due to limited availability and long travelling distances [1, 2]. This holds also true for treatment of acute stroke patients where treatment delays are associated with worse outcome [3–5]. Approved treatments for acute stroke include intravenous thrombolysis or mechanical revascularization which may improve stroke symptoms. However, these procedures are only effective within a certain time frame [6–8]. Thus, different efforts have been made to shorten the door-to-needle-time (DTNT) by optimizing in-hospital processes [9, 10]. To reduce the onset-to-door-time (OTDT), however, educational means among the population are needed as well as changes in the pre-hospital admission processes to the nearest qualified hospital. While the first can increase the awareness for stroke signs and for immediate action in the general public, the implementation of telestroke units can reduce the spatial distance to a facility providing help and, thus, avoid time elapsing senselessly [11, 12].

Previous studies from Europe and the United States showed differences between urban and rural regions in terms of stroke incidence as well as management after a cerebrovascular event [13, 14] resulting in a higher stroke mortality in rural regions compared to urban areas [15]. In addition, a lower awareness and recognition of stroke symptoms as well as of stroke risk factors can be found in the rural population [16, 17]. Finally, an insufficient training of paramedics in pre-hospital stroke management and considerable delays in triage of stroke patients as well as diagnostic testing and a lack of experience in intravenous thrombolysis have to be counted among the reasons for urban-rural disparities [18].

### Telemedical networks

In order to address the challenge of minimizing rural/urban differences and providing appropriate health care independent of population density, telemedicine networks were proposed for providing expert support and for bridging long distances by audio-visual means [19, 20]. In terms of stroke care this means a 24/7-service for non-specialised community hospitals provided by hospitals with expertise in stroke care [21]. In Germany, during the last two decades telemedical networks have been established including academic and community hospitals in sparsely-populated regions [21]. While positive impacts have been demonstrated [22, 23], most reports, however, focus on one specific issue, such as the reliable determination of the NIHSS score [24], a timely access to computer tomography [25] or to intravenous thrombolysis [9, 26–30].

### Aims

On the basis of a telemedical stroke network comprising all hospitals in north-western Bavaria (Germany), we aimed to evaluate the impact of the network structure on stroke care in a mainly rural area. Over the first 2 years, the development of a set of predefined health care quality indicators covering aspects of diagnostics, therapy and stroke outcome was analysed in detail.

## Methods

### Study region and structure of the network

The *Tra*nsregional *N*etwork for *S*troke *I*ntervention with *T*elemedicine (TRANSIT-Stroke) was established in October 2014. It covers an area of about 10,500 km^2^ in north-western Bavaria, comprising Lower Franconia and two neighbouring districts with a catchment area of 1.5 million inhabitants (census 12/2017). According to the NUTS3 definition of the European Union, two-thirds of the area are classified as ‘predominantly rural’ [31] with a population density of 97 inhabitant per km^2^.

The TRANSIT-Stroke Network currently comprises a total of 12 hospitals, comprising three levels of expertise in stroke care (status as at 31.03.2019):

- Level-I-hospitals (*n* = 6) provide an intermediate care unit but have no certified stroke unit. They are able to conduct CT and CT-angiography and, thus, intravenous thrombolysis.
- Level-II-hospitals (*n* = 2) provide a regional stroke unit which is certified by the German Stroke Society (DSG) [32]. These stroke units comprise at least four beds and treat at least 250 stroke patients per year. Beside CT and CT-angiography, neuroradiological services are available during regular working-hours and on call.
- Level-III-hospitals (*n* = 4) provide a supra-regional stroke unit certified according to German Stroke Society (DSG), treating at least 500 stroke patients per year. The stroke unit encompasses at least 6 beds. As stroke centres, they allocate access to all relevant therapeutic and diagnostic facilities as well as services of a neurosurgery and an interventional neuroradiology [33, 34].

With these 12 clinical centres in the network, TRANSIT-Stroke comprises all hospitals offering stroke care within north-western Bavaria and its surroundings. The involvement of all three levels of stroke care is a unique feature within networks located in Germany. A recently published analysis showed, that level-I-hospitals are situated predominantly at the edge of a 30-min-accessibility of level-III-hospitals [35].

Within the network, there is a two-sided interaction between the levels of care. While three of the stroke centres (level III) provide teleconsultations for hospitals of level I and II, the latter can transfer patients requiring specialized care to hospitals of higher levels. In addition, specific training options for personnel in level-I-hospitals are provided by specialized personnel of level-III-hospitals. These included additional teleconsultations focusing on secondary prevention once a month as well as on on-site visits twice a year. Professional face-to-face training courses took place twice a year, at least, in which stroke specific knowledge was taught, such as surveying the NIHSS. During teleconsultations, guidance for conducting intravenous thrombolysis (IVT; dissolving the drug, preparing infusion, administering IVT) was given in case of need step by step. Regarding transferral to another hospital, decision was made by level-III-experts depending on brain image findings, suspected stroke aetiology, and severity of symptoms.. Beside this vertical exchange, there is also a horizontal integration in respect to a lack of specific treatment facilities (i.e., neurosurgery, interventional neuroradiology or intensive care) in level-II/III-hospitals.

### Data collection

Data on quality of acute hospital care are collected within the regional stroke register Bavaria, a member of the German Stroke Registers Study Group (ADSR) [36]. For patients with a transient ischaemic attack (TIA) (ICD-10: G45) or an ischaemic, haemorrhagic or undefined stroke (ICD-10: I61, I63, I64) a predefined set of data was recorded. Data collection encompassed information on the index event as well as on diagnostics, treatment and outcome. In addition, information on co-morbidities and risk factors as well as on complications and discharge were documented. Data collection is mandatory since 2013. Information was recorded digitally by members of the staff at each hospital and transferred electronically to the coordinating centre of the Bavarian Permanent Working Party for Quality Assurance in Munich, Germany. Completeness of data collection is checked electronically, but not monitored with regard to content. Every participating hospital contributed data for internal analyses. For the present study, however, we included only those hospitals, which were actively involved in the network and delivered data for the whole study period (i.e., 2015 and 2016). Thus, data of eight hospitals were available for this analysis (three level-I-, two level-II- and three level-III-hospitals). The number of teleconsultations conducted per hospital was recorded on a monthly basis.

### Quality indicators (QI)

In order to measure quality of stroke care, a set of evidence-based quality indicators (QI) was defined covering different aspects of stroke care in the following domains: diagnostics, treatment and outcome [36]. These indicators were developed by the multidisciplinary quality indicator board of the German Stroke Register Study group within a standardized process comprising a literature review, a standardized consensus procedure, an external evaluation and a pilot study [36]. The set of quality indicator is updated regularly. For the present analysis, the following QIs covering different processes of stroke care were analysed [23] using their target values of 2016 (see also Table 2): Brain imaging (CT or MRI) within 60 min after admission, vascular imaging (ultrasonography, CT-angiography or MR-angiography) within 48 h after admission, intravenous thrombolysis in patients aged 18–80 years with symptom onset within 4 h and an NIHSS score of ≤25 points, treatment with platelet inhibitor ≤48 h after onset in patients aged ≥18 years, treatment with platelet inhibitor at discharge in patients aged ≥18 years, atrial fibrillation screening in stroke patients without known AF, dysphagia screening within 2 days after admission, mobilization within 2 days after admission, physiotherapy within 2 days after admission, speech therapy within 2 days after admission, rehabilitation assessment in handicapped stroke patients, antihypertensive drug prescription in hypertensive patients, prescription of statins at discharge, door-to-needle-time, and revascularisation of symptomatic carotid stenosis (definition of each single item is provided in the online supplement (see additional file 1)). Target values indicating good quality of care were defined by the quality indicator board of the German Stroke Register Study group.

### Statistical analysis

Statistical analyses were stratified for levels of hospitals (level-I- vs. level-II/III-hospitals). Data are presented as absolute and relative frequencies as well as mean ± standard deviation (SD) or – where appropriate – as median and interquartile range (IQR). For each QI the percentage of patients fulfilling it was calculated quarterly and put in relation to the respective predefined threshold. Time trends in performance for quarters of 2 years were investigated with (asymptotic) Cochrane-Armitage-Test. The level of significance was set to alpha = 0.05. All analyses were performed using SAS 9.4.

### Ethics

The TRANSIT-Stroke network and data acquisition has been approved by the Ethic Committee of the University of Würzburg (54/14) and was registered in the German Registry for Clinical Studies (DRKS: No. 11696). The data presented were collected for the purpose of quality assurance and, thus, the identity of the individual patients were anonymous. Therefore, no specific informed consent on individual level was obtained by the patients.

## Results

Overall, information on 7881 patients was included in the evaluation, of which 927 (12%) were treated in level-I-hospitals. Given in total and stratified by hospital level, the distributions in patient characteristics of diagnostics and treatments are displayed in Table 1. Between 01/2015 and 12/2016, a total of 1896 teleconsultations were conducted in the examined hospitals, whereof 1542 (81%) have been in interaction with hospitals of level I.

**Table 1:** Description of the study population, total as well as stratified for levels of hospitals

			total		level-I-hospitals		level-II/III-hospitals		*p*-value of levels
			*n* = 7881		*n* = 927		*n* = 6954		
age									<.0001
years		mean (SD)	74.6	(12.8)	76.42	(12.2)	74.36	(12.9)	
sex									0.0029
male		n(%)	4070	(51.6)	436	(47.0)	3634	(52.3)	
female		n(%)	3811	(48.4)	491	(53.0)	3320	(47.7)	
diagnosis									<.0001
transient ischaemic attack	TIA	n(%)	1938	(24.6)	334	(36.0)	1604	(23.1)	
ischaemic stroke	IS	n(%)	5409	(68.6)	527	(56.9)	4882	(70.2)	
haemorrhagic stroke	HS	n(%)	504	(6.4)	44	(4.8)	460	(6.6)	
undefined stroke	US	n(%)	30	(0.4)	22	(2.4)	8	(0.1)	
treatment									
^#^lyse iv		n(%)	1113	(20.6)	50	(9.5)	1063	(21.8)	<.0001
^#^lyse ia		n(%)	68	(1.3)	1	(0.2)	67	(1.4)	0.0206
^#^thrombectomy		n(%)	226	(4.2)	2	(0.4)	224	(4.6)	<.0001
ventilation		n(%)	378	(4.8)	25	(2.7)	353	(5.1)	0.0014
diagnostics									
dysphagia screening		n(%)	6895	(87.5)	396	(42.7)	6499	(93.5)	<.0001
long-term ECG		n(%)	7206	(91.4)	844	(91.1)	6362	(91.5)	0.6134
extracranial		n(%)	7457	(94.6)	780	(84.1)	6677	(96.0)	<.0001
< 48 h		n(%)	7003	(88.9)	583	(62.9)	6420	(92.3)	
> = 48 h		n(%)	454	(5.8)	197	(21.3)	257	(3.7)	
intracranial		n(%)	7223	(91.7)	571	(61.6)	6652	(95.7)	<.0001
< 48 h		n(%)	6939	(88.1)	548	(59.1)	6391	(91.9)	
> = 48 h		n(%)	284	(3.6)	23	(2.5)	261	(3.8)	
NIHSS – at admission									0.0290
		median (IQR)	3	(1–7)	3	(1–6)	3	(1–7)	
0		n(%)	1343	(17.1)	168	(18.1)	1175	(16.9)	
1–4		n(%)	3643	(46.2)	406	(43.8)	3237	(46.6)	
5–15		n(%)	2227	(28.3)	289	(31.2)	1938	(27.89)	
16–20		n(%)	371	(4.7)	29	(3.1)	342	(4.9)	
21–42		n(%)	294	(3.7)	35	(3.8)	259	(3.7)	
mRS – at admission									
		median (IQR)	3	(1–4)	3	(1–4)	3	(1–4)	
0–1		n(%)	2191	(27.8)	267	(28.8)	1924	(27.7)	0.4751
2–6		n(%)	5686	(72.2)	660	(71.2)	5026	(72.3)	
0–2		n(%)	3809	(48.4)	454	(49.0)	3355	(48.3)	0.6880
3–6		n(%)	4068	(51.6)	473	(51.0)	3595	(51.7)	
mRS – at discharge									
		median (IQR)	2	(0–3)	2	(0–3)	2	(0–3)	
0–1		n(%)	3616	(45.9)	453	(48.9)	3163	(45.5)	0.0550
2–6		n(%)	4259	(54.0)	474	(51.1)	3785	(54.5)	
0–2		n(%)	5170	(65.7)	670	(72.3)	4500	(64.8)	<.0001
3–6		n(%)	2705	(34.4)	257	(27.7)	2448	(35.2)	
Barthel Index – at admission									0.0003
		mean (SD)	62.50	(35.1)	60.81	(34.3)	62.72	(35.2)	
		median (IQR)	75.00	(37.5–100)	52.50	(37.5–100)	75.00	(37.5–100)	
Barthel Index – at discharge									<.0001
		mean (SD)	73.28	(32.7)	75.07	(29.8)	73.05	(33.1)	
		median (IQR)	87.50	(50.5–100)	87.50	(62.5–100)	87.50	(50–100)	
Comorbidities									
diabetes		n(%)	2038	(25.9)	255	(27.5)	1783	(25.6)	0.2243
hyper tonus		n(%)	6798	(86.3)	805	(86.8)	5993	(86.2)	0.5983
atrial fibrillation		n(%)	2301	(29.2)	263	(28.4)	2038	(29.3)	0.8371
previous stroke		n(%)	2269	(28.8)	238	(25.7)	2031	(29.2)	0.0253
onset-to-door-time									<.0001
< =1 h		n(%)	892	(11.3)	63	(6.8)	829	(11.9)	
1-2 h		n(%)	1436	(18.2)	144	(15.5)	1292	(18.6)	
2-3 h		n(%)	780	(9.9)	94	(10.1)	686	(9.9)	
3-3.5 h		n(%)	257	(3.3)	48	(5.2)	209	(3.0)	
3.5-4 h		n(%)	238	(3.0)	34	(3.7)	204	(2.9)	
4-6 h		n(%)	562	(7.1)	77	(8.3)	485	(7.0)	
6-24 h		n(%)	1383	(17.6)	189	(20.4)	1194	(17.2)	
24-48 h		n(%)	564	(7.12)	85	(9.2)	479	(6.9)	
> 48 h		n(%)	812	(10.3)	58	(6.3)	754	(10.9)	
unknown		n(%)	953	(12.1)	135	(14.6)	818	(11.8)	
^#^door-to-needle-time									<.0001
< =30 min		n(%)	619	(11.5)	4	(0.8)	615	(12.6)	
30-60 min		n(%)	409	(7.6)	29	(5.5)	380	(7.8)	
> 60 min		n(%)	147	(2.7)	17	(3.2)	130	(2.7)	
no lysis applied		n(%)	4232	(78.3)	477	(90.5)	3755	(77.0)	
length of stay									0.1129
days		mean (SD)	8.5	(6.3)	8.8	(6.4)	8.4	(6.3)	
secondary prevention									
anticoagulation		n(%)	1999	(25.4)	244	(26.3)	1755	(25.2)	0.4836
VitKAnt		n(%)	923	(11.7)	130	(14.0)	793	(11.4)	
NOAK		n(%)	1076	(13.7)	114	(12.3)	962	(13.8)	
statins		n(%)	5827	(73.9)	763	(82.3)	5064	(72.8)	<.0001
IPA within 48 h		n(%)	6053	(76.8)	770	(83.1)	5283	(76.0)	<.0001
IPA at discharge		n(%)	5534	(70.2)	673	(72.6)	4861	(69.9)	0.0281
in-hospital mortality									0.4158
		n(%)	423	(5.4)	55	(5.9)	568	(5.3)	
reason of discharge									<.0001
end of treatment as scheduled		n(%)	4938	(62.9)	729	(78.6)	4209	(60.6)	
end of treatment due to other reason		n(%)	123	(1.6)	14	(1.5)	109	(1.6)	
transfer to other hospital		n(%)	477	(6.1)	46	(5.0)	431	(6.2)	
transfer to acute rehabilitation		n(%)	1358	(17.2)	68	(7.3)	1290	(18.6)	
transfer to skilled nursing facility		n(%)	455	(5.8)	13	(1.4)	442	(6.4)	
transfer to other department		n(%)	107	(1.4)	2	(0.2)	105	(1.5)	
death		n(%)	420	(5.3)	55	(5.9)	365	(5.3)	

^#^ as basis only ischaemic stroke is taken, i.e. 5409 = 527 + 4882

In level-I-hospitals, patients were older and more often female compared to level-II/II-hospitals. In level-I-hospitals, more TIAs but less ischemic strokes were diagnosed compared to level-II/III-hospitals during the whole study period (Table 1).

In level-II/III-hospitals, adherence to QIs was constantly high ab initio (Table 2). Ten QIs out of 15 reported clearly surpassed the target value in every quarter of 2015 and 2016. In five QIs, observed values oscillated closely around the target value.

**Table 2:** Indicators of quality displayed quarterly, stratified for level of hospital, patients [%] treated as defined

	2015				2016				trend *p*-value	target value
	Q 1	Q 2	Q 3	Q 4	Q 1	Q 2	Q 3	Q 4		
**Early cerebral imaging (< 60 min after admission) in patients eligible for thrombolysis**										70
level-I	47.8	40.9	60.0	48.3	54.6	67.7	60.7	60.0	0.0884	
level-II/III	75.1	73.3	75.5	71.9	72.9	71.1	70.6	66.0	0.0159	
all levels	72.9	70.5	74.3	69.5	70.3	70.7	69.5	65.3	0.0569	
**Door-to-needle-time < 60 min in patients with IVT**										90
level-I	100.0	100.0	57.1	55.6	60.0	42.9	57.1	90.9	0.6475	
level-II/III	91.8	88.6	87.3	84.7	92.3	91.0	94.8	86.2	0.8483	
all levels	91.9	88.9	85.8	82.9	91.0	88.4	93.0	86.5	0.9491	
**Early intravenous thrombolysis (IVT) given in patients with indication for IVT**										35
level-I	0.0	10.0	8.3	20.0	13.3	35.7	28.6	44.4	0.0015	
level-II/-III	40.9	43.1	47.3	39.7	40.5	45.6	45.7	41.8	0.7612	
all levels	38.9	40.8	44.3	37.9	37.5	44.8	44.1	42.1	0.4222	
**Platelet inhibitor given within 48 h in patients with IS or TIA**										95
level-I	96.4	96.7	98.3	93.0	98.6	98.7	100.0	100.0	0.0295	
level-II/III	97.0	96.1	93.0	96.1	93.7	93.3	94.4	93.2	0.0074	
all levels	96.9	96.1	93.6	95.8	94.3	94.1	95.1	94.2	0.0543	
**Dysphagia screening**										90
level-I	26.3	33.3	56.1	35.7	32.4	55.7	58.2	59.1	<.0001	
level-II/III	95.6	97.9	96.6	95.0	98.1	97.5	96.2	96.0	0.9489	
all levels	91.5	92.6	92.9	90.0	90.8	93.7	91.6	92.4	0.7562	
**Early speech and language therapy for patients with dysphagia / dysphasia / dysarthria**										80
level-I	63.2	58.6	85.0	65.4	61.8	97.0	72.3	73.2	0.2191	
level-II/III	95.9	98.2	92.0	96.0	97.6	96.3	96.3	95.2	0.9342	
all levels	94.2	94.9	91.2	93.7	93.8	96.4	93.2	92.7	0.8144	
**Early physio- / occupational therapy for patients with motor disability**										90
level-I	66.7	76.9	90.3	95.2	82.1	87.5	97.5	84.2	0.0714	
level-II/III	97.8	97.8	97.1	98.6	97.4	98.8	96.3	97.4	0.5997	
all levels	96.4	95.7	96.3	98.3	96.0	97.4	96.5	95.7	0.9579	
**Early mobilisation of patients with severe disability**										90
level-I	96.7	90.7	98.1	100.0	96.2	94.6	95.9	100.0	0.3319	
level-II/III	97.8	98.0	95.9	97.2	97.1	98.2	95.7	94.8	0.0216	
all levels	97.7	97.2	96.1	97.5	97.0	97.8	95.7	95.4	0.0645	
**Patients receiving an atrial fibrillation screening during their stay**										80
level-I	87.5	88.4	93.3	91.4	91.0	88.3	88.3	96.3	0.2601	
level-II/III	92.1	93.0	92.3	92.8	94.0	92.8	94.9	94.1	0.0435	
all levels	91.8	92.4	92.5	92.6	93.6	92.2	94.0	94.4	0.0312	
**Extracranial carotid artery diagnostic in patients with IS or TIA**										80
level-I	77.4	69.2	78.6	75.5	80.0	87.9	88.1	90.8	<.0001	
level-II/III	92.4	94.9	94.1	95.8	96.7	95.7	94.5	95.0	0.0295	
all levels	91.4	92.0	92.3	93.8	94.5	94.7	93.6	94.4	0.0006	
**Revascularisation of symptomatic carotid stenosis in patients with IS or TIA**										60
level-I	0.0	100.0	–	–	50.0	100.0	100.0	66.7	0.4174	
level-II/III	60.0	41.2	62.5	54.2	52.6	57.1	72.7	61.5	0.3310	
all levels	57.7	44.4	62.5	54.2	52.4	62.5	73.9	62.1	0.2315	
**Antihypertensive drugs at discharge in patients with IS or TIA**										95
level-I	94.9	98.7	100.0	98.6	98.2	99.0	99.1	99.1	0.3111	
level-II/III	97.0	97.4	97.4	97.3	98.2	95.7	96.7	96.7	0.2404	
all levels	96.9	97.5	97.7	97.4	98.2	96.2	97.1	97.0	0.4743	
**Patients receiving statin at discharge or whom a statin was recommended**										80
level-I	68.4	69.4	90.2	89.9	89.2	86.6	95.7	93.6	<.0001	
level-II/III	71.5	79.7	77.4	76.9	80.3	78.8	79.8	77.6	0.0069	
all levels	71.3	78.5	78.8	78.2	81.5	79.8	82.0	79.7	<.0001	
**Platelet inhibitor given at discharge in patients with IS or TIA and no anticoagulation**										95
level-I	88.6	89.5	97.1	95.7	95.2	95.4	100.0	94.6	0.0302	
level-II/III	97.6	97.0	95.9	94.7	95.8	94.8	95.9	94.7	0.0101	
all levels	97.1	96.1	96.0	94.8	95.8	94.9	96.4	94.7	0.0101	
**Discharge in rehabilitation clinic**										70
level-I	38.5	66.0	61.0	54.8	52.2	70.0	81.3	77.6	0.0005	
level-II/III	75.3	77.9	78.1	77.1	77.1	80.6	78.3	76.2	0.6075	
all levels	72.7	76.4	76.2	75.3	73.9	79.4	78.7	76.3	0.1302	

**Fig. 1:**
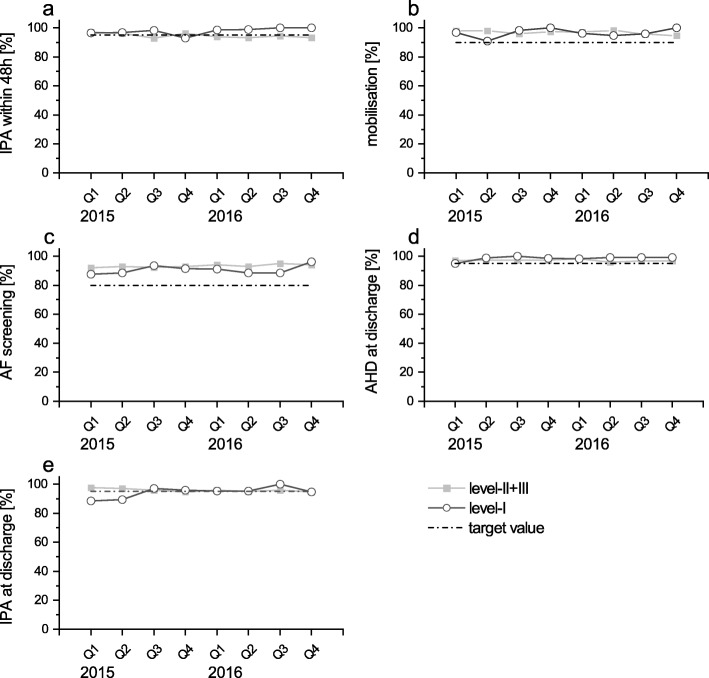
Indicators of quality high ab initio, displayed quarterly, stratified for level of hospital, patients [%] treated as defined. a) Platelet inhibitor given within 48 h in patients with IS or TIA. b) Early mobilisation of patients with severe disability. c) Patients receiving an atrial fibrillation screening during their stay. d) Antihypertensive drugs at discharge in patients with IS or TIA. e) Platelet inhibitor given at discharge in patients with IS or TIA and no anticoagulation

In level-I-hospitals, observations were more heterogeneous (Table 2). A total of five QIs surpassed the target value ab initio (percentage of patients receiving early mobilisation, atrial fibrillation screening, antihypertensive drugs therapy) (Fig. 1). Their values remained constant over the whole study period. Prescription of inhibitors of platelet aggregation was close to the requested threshold as well, irrespective of whether they were prescribed within 48 h after onset of stroke or at discharge. QIs showing a high adherence ab initio mainly comprised measures of secondary stroke prevention.

**Fig. 2:**
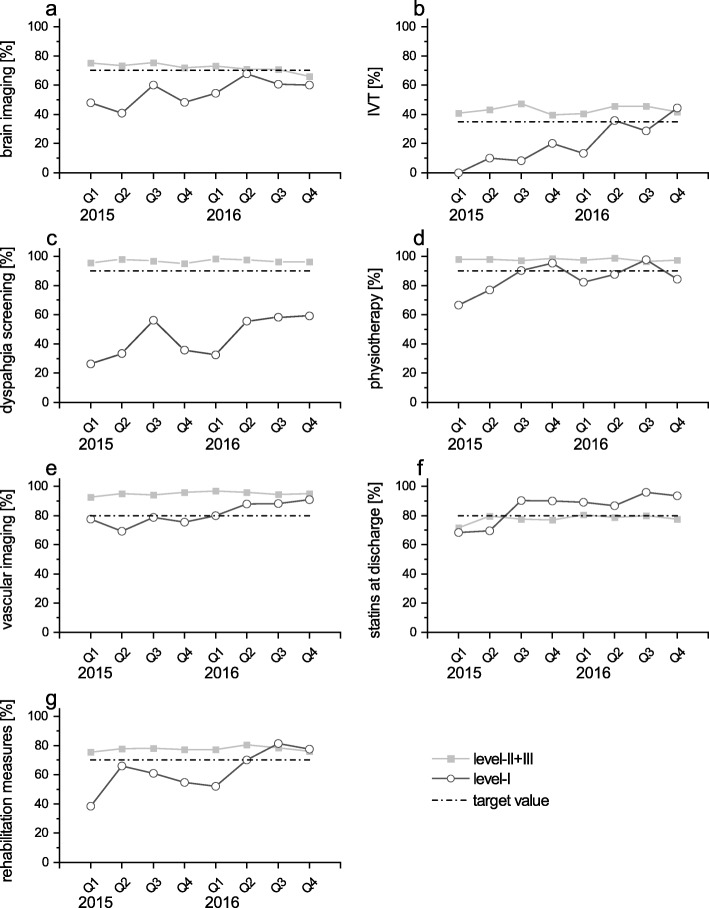
Indicators of quality increasing over time, displayed quarterly, stratified for level of hospital, patients [%] treated as defined. a) Early cerebral imaging (< 60 min after admission) in patients eligible for thrombolysis. b) Early intravenous thrombolysis (IVT) given in patients with indication for IVT. c) Dysphagia screening. d) Early physio- / occupational therapy for patients with motor disability. e) Extracranial carotid artery diagnostic in patients with IS or TIA. f) Patients receiving statin at discharge or whom a statin was recommended. g) Discharge in rehabilitation clinic

In seven QIs a continuous improvement was observed in level-I-hospitals during the study period. Vascular imaging as well as prescription of statins were raised in level-I-hospitals within the first three quarters of 2015 (trend *p* < .0001 and p < .0001, respectively), surpassing the required threshold and, thereafter, keeping high values (Fig. 2: e, f). Application of physiotherapy was enhanced at an early stage, but then stabilized oscillating around the given threshold (trend *p* = 0.07) (Fig. 2: d). Implementation of intravenous thrombolysis and transfer to rehabilitation measures proceeded slowly and the required threshold was achieved late (Fig. 2: b, g). In both indices the observed values surpassed the predefined target values only within the last three quarters of 2016. The trend, however, was highly significant (trend *p* = 0.0015 and *p* = 0.0005, respectively). Screening for dysphagia as well as brain imaging also exhibited a positive trend (trend *p* < .0001 and *p* = 0.09, respectively), both items did not reach the required threshold within the study time (Fig. 2: a, c). Indices of quality showing an improvement over time were predominantly related to stroke specific diagnostics and in-hospital organisation.

**Fig. 3:**
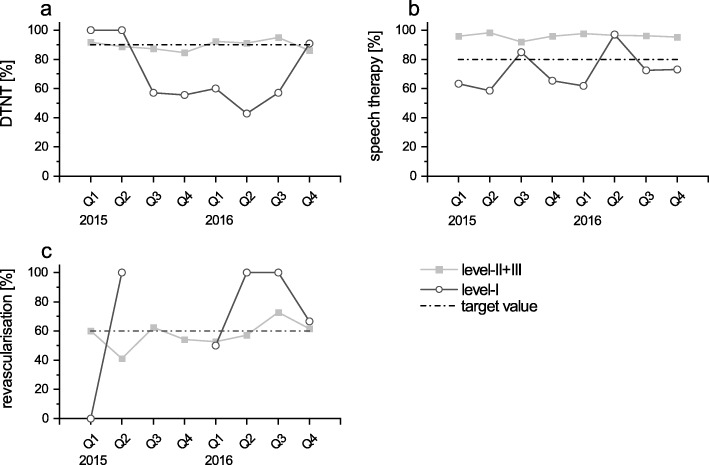
Indicators of quality with no clear temporal trend, displayed quarterly, stratified for level of hospital, patients [%] treated as defined. a) Door-to-needle-time <  60 min in patients with IVT. b) Early speech and language therapy for patients with dysphagia / dysphasia / dysarthria. c) Revascularisation of symptomatic carotid stenosis in patients with IS or TIA

No clear temporal trend was observed in three QIs in level-I-hospitals (Fig. 3). Values related to the implementation of speech therapy within the first 48 h oscillated around the threshold value, but did not demonstrate a clear development towards an increased rate (trend *p* = 0.22). Values regarding the conduction of revascularisation within the first 14 days after onset of stroke as well as the percentage of patients having a door-to-needle-time of less than 60 min varied tremendously from quarter to quarter. Of note, data of the latter indicators were available for less than 15% of the total study population. Thus, especially the number of cases reported in level-I-hospitals are very low, i.e. single-digit (results not shown). Indices of quality showing no clear time trends can be grouped as therapeutic measures.

## Discussion

At the end of the first 2 years after establishment of the TRANSIT-Stroke Network in north-western Bavaria, stroke patients received a treatment according to the predefined standards or even better in 10 out of 15 QIs analysed. In level-II/III-hospitals, adherence to target values was constantly high ab initio. In level-I-hospitals, 10 out of 15 QIs surpassed the thresholds of quality of care within the first 2 years after establishing the TRANSIT-Stroke Network. Thus, stroke care improved in level-I-hospitals since TRANSIT-Stroke was launched.

### Population

Patients assigned to level-I-hospitals were slightly older compared to those assigned to level-II/III-hospitals. This is in line with other studies evaluating urban-rural differences in stroke care [37–39]. However, there are also reports on balanced distribution [15, 18] or younger patients in level-I-hospitals [28, 40]. We observed a higher portion of women in level-I-hospitals while in level-II/III-hospitals the distribution was reversed. This opposite direction of the distribution between the sexes has not been observed yet. Though other studies also report different proportions of sexes in different hospital levels, the majority clearly stays with one sex [15, 18, 28, 38, 40].

While patients with ischaemic or haemorrhagic stroke were transferred to specialized clinical centres, i.e. level-II/III-hospitals, patients with TIA (ICD-10: G 45) were more frequently treated in level-I-hospitals. The higher proportion of unspecified strokes (ICD-10: I 64) in level-I-hospitals might point out their missing resources to determine the aetiology of stroke. The difference of diagnoses found in stroke centres and in communal hospitals in TRANSIT-Stroke Network was comparable to that in other studies [18, 37]. Of note, this difference appeared irrespectively whether the analysed hospitals were organised in a network [37] or not [18].

In the observed period, a total of 1542 teleconsultations were performed in interaction with level-I-hospitals, of which 927 (60%) were strokes and TIAs and, thus, 615 (40%) were mimics or other diseases. This share is consistent with – though much higher than – other networks reporting 20% of mimics [25].

### Quality indicators

#### Intravenous thrombolysis

In the first year after implementation, an increase from 0 to 20% was observed regarding intravenous thrombolysis in eligible patients. The rate of thrombolysis has been more than doubled in the second year of data assessment, achieving a rate of 44% in level-I-hospitals at the end of the analysed period. This finding was similar to other German telestroke networks [23].

The rate of thrombolysis in the TRANSIT-Stroke Network is not directly comparable to previous studies, as the denominator was restricted to selected patients [21, 23, 39]. Our results support the observation of other telestroke networks, that telemedicine enables comprehensively state-of-the-art treatments [19, 24, 26]. However, diminishing of urban-rural disparities in the use of thrombolysis over time can be observed independently from the established network. In Canada, rural hospitals also extended their medical services regarding thrombolysis and, thus, reduced distance to urban hospitals within a decade [18], without being organised in a network.

#### Secondary prevention

Whereas most studies focus on short- and long-term outcomes after thrombolysis recommended via teleconsultation [22, 26, 30, 41], reports on changes in prescribing secondary prevention following teleconsultation are sparse. In TRANSIT-Stroke, prescription of antiplatelet and antihypertension drugs was high ab initio in both, level-I- and level-II/III-hospitals (on average 95 and 98%, respectively). Studies in Canada and the US [18, 40] corroborate our results regarding antiplatelet drugs but, overall, the percentages of patients receiving antiplatelet therapy was lower (80%). This was also true for antihypertensive drugs (73%) [40]. Additionally, the rate in prescribing statins improved in level-I-hospitals significantly with the establishment of TRANSIT-Stroke. Values in the beginning of the study period were comparable to that of rural regions not being part of a network [18, 40]. At the end of the observed period, level-I-hospitals exceeded the predefined treatment targets and even surpassed level-II/III-hospitals.

#### Diagnostic examinations after stroke

Implementation of diagnostic examinations, i.e., brain and vascular imaging as well as dysphagia screening, increased in level-I-hospitals within the first 2 years in the present network. Results for brain imaging reported elsewhere are not comparable since the selected time window from admission to brain imaging was much longer than in the present study (1 h [21] and 24 h [38] vs. 30 min). However, studies reported a lower number of patients receiving brain imaging in rural areas [38] and have shown that being part of a telestroke network enhanced its utilisation [21]. In respect to carotid imaging, our results exceeded those reported for rural areas in Canada [18] and corresponded with those found in other networks in Germany [21]. Dysphagia screening was performed in 26% of all patients in level-I-hospitals and, thus, exceeded observations made in rural areas in the US (17% [39]) ab initio. However, development within 2 years after implementing TRANSIT-Stroke has neither reached the predefined target values nor values observed in another German telestroke network [21].

#### Rehabilitative measures

Reports on disparities in rehabilitative measures are sparse in literature. Our findings in level-I-hospitals regarding physiotherapy, speech therapy, occupational therapy and inpatient rehabilitation after discharge exceed those of rural and even of urban areas in the US [18] and were comparable to those in another German telestroke network [21].

### Telemedical networks

While other networks have hubs and spoke, i.e. two levels of hospitals [23, 41, 42], TRANSIT-Stroke involves all three levels currently defined in Germany. Thereby, all these hospitals are integrated in the network and, thus, in telemedical counselling and in regular training courses. Especially these regular training options within TRANSIT-Stroke are thought to be the reason for the improvement of several QIs in level-I-hospitals.

### Strengths and limitations

The present study has several strengths: The QIs presented in this analysis cover all sections of in-house treatment and describe stroke care extensively. By including different QIs regarding diagnostics, secondary prevention and cerebrovascular imaging, we were able to increase knowledge about issues which have not been investigated well so far; some QIs were even reported for the first time. We are aware of the following limitations: Our results may be biased due to the limited numbers of patients admitted to level-I-hospitals, representing 12% of the population included in the analysis. This distribution is due to the setting of rather low population density and comparable to other studies investigating urban and rural health care (range from 7 to 16%) [14, 18, 39, 40, 43]. In addition, not all hospitals participating in the network to date were included in the present analysis. Due to reasons of privacy protection in Germany, transferral within the TRANSIT-Stroke Network could not be considered for analyses. The QIs used in this analysis focused on in-house aspects of stroke care. Thus, issues such as awareness of stroke in the population, reduction of onset-to-door-time before and after implementation of the network or quality of stroke rehabilitation stay disregarded. Assessment of treatment safety, e.g., regular monitoring of haemorrhagic complications (asymptomatic/symptomatic intracranial haemorrhages) was not part of this analysis. However, newly defined quality indicators will be included in further studies to improve additionally health care and, thus, for example, will also consider mechanical thrombectomy. Implausibilities at first sight in the descriptive data, such as reported thrombectomies in level-I-hospitals, were not monitored but can be explained by re-transfer after surgery.

With the implementation of the TRANSIT-Stroke Network the range of specialists’ expertise is extended beyond the 30-min-accessibility of level-II- and level-III-hospitals. Thereby, it is delivered towards rural areas without time delay caused by transportation.

## Conclusion

In conclusion, the implementation of a comprehensive telestroke network in north-western Bavaria raised the quality of care in level-I-hospitals and resulted in an improvement of early stroke-specific diagnostics, inpatient rehabilitative measures and an improvement of secondary prevention.

## Supplementary information

**Additional file 1.** Brief description of quality indicators applied in this analysis. Definitions of the German quality indicators in acute stroke care as defined by the Quality Indicator Board of the German Stroke Registers Study Group.

## Data Availability

The data that support the findings of this study are available from Bavarian Permanent Working Party for Quality Assurance in Munich, Germany [Bayerische Arbeitsgemeinschaft Qualitätssicherung (BAQ)] but restrictions apply to the availability of these data, which were used under license for the current study, and so are not publicly available. Data are, however, available from the authors upon reasonable request and with permission of Bavarian Permanent Working Party for Quality Assurance in Munich, Germany [Bayerische Arbeitsgemeinschaft Qualitätssicherung (BAQ)].

## Abbreviations

ADSR: Arbeitsgemeinschaft Deutschsprachiger Schlaganfall-Register (German Stroke Registers Study Group)
BAQ: Bayerische Arbeitsgemeinschaft für Qualitätssicherung (Bavarian Permanent Working Party for Quality Assurance)
CT: Computer tomography
DRKS: Deutsches Register Klinischer Studien (German Registry for Clinical Studies)
DSG: Deutsche Schlaganfall-Gesellschaft (German Stroke Society)
DTNT: Door-to-needle-time
HS: Haemorrhagic stroke
ICD-10: International Statistical Classification of Diseases and Related Health Problems, 10^th^ Revision
IQR: Interquartile range
IS: Ischaemic stroke
MRI: Magnetic resonance imaging
NIHSS: National Institute of Health Stroke Scale
NUTS 3: Nomenclature of Territorial Units for Statistics, district level
OTDT: Onset-to-door-time
QI: Quality Indicator
SD: Standard deviation
SU: Stroke Unit
TIA: Transient ischaemic attack
TRANSIT: Transregional Network for Stroke Intervention with Telemedicine
US: Undefined stroke
