# Supplementary material for: Two years’ experience of implementing a comprehensive telemedical stroke network comprising in mainly rural region: the Transregional Network for Stroke Intervention with Telemedicine (TRANSIT-Stroke)
Source: BMC Neurol. 2020 Mar 19;20:104. doi: 10.1186/s12883-020-01676-6 (PMC7081707; doi:10.1186/s12883-020-01676-6)
Supplement: Supplementary file 1 — Additional file 1. Brief description of quality indicators applied in this analysis. Definitions of the German quality indicators in acute stroke care as defined by the Quality Indicator Board of the German Stroke Registers Study Group. [file 12883_2020_1676_MOESM1_ESM.pdf]

Additional file 1: Definitions of the German quality indicators in acute stroke care as defined by the Quality Indicator Board of the German Stroke Registers Study Group.

	Brief description	target value (2016)
brain imaging	**Early cerebral imaging (< 60 min after admission) in patients eligible for thrombolysis**<br>Numerator: Patients with cerebral imaging (CCT/MRI) within the first hour of admission.<br>Denominator: All patients within the age range 18-80 and with onset-admission ≤ 2 h and NIHSS of 4-25.	70 %
door-to-needle-time (DTNT)	**Door-to-needle-time < 60 min in patients with IVT**<br>Numerator: Patients with door-to-needle time <60 minutes.<br>Denominator: Patients with cerebral infarction and IVT.	90 %
intravenous thrombolysis (IVT)	**Early intravenous thrombolysis (IVT) given in patients with indication for IVT**<br>Numerator: Patients that received intravenous thrombolysis (IVT).<br>Denominator: Patients with cerebral infarction, age 18-80 and with onset-admission ≤ 2 h and NIHSS 4-25. Patients with intra-arterial thrombolysis are excluded.	35 %
platelet inhibitor ≤ 48 h	**Platelet inhibitor given within 48 h in patients with IS or TIA**<br>Numerator: All patients that were given platelet inhibition within 48h after onset.<br>Denominator: Denominator: All patients with TIA and cerebral infarction. Patients that receive anticoagulation or where onset to admission is >48h are excluded as well as those aged <18 and those discharged, transferred or died on the first day.	95 %
dysphagia screening	**Dysphagia screening**<br>Numerator: Patients with dysphagia screening per protocol.<br>Denominator: All patients with stroke. Patients with TIA, impaired consciousness, or those where dysphagia screening was not possible or those that were discharged/transferred or died on the day of admission are excluded.	90 %
speech therapy	**Early speech and language therapy for patients with dysphagia / dysphasia / dysarthria**<br>Numerator: Patients that received treatment by speech and language therapy within the first 2 days.<br>Denominator: All patients with dysphasia/dysarthria/dysphagia. Patients with TIA, those unconscious and those that were discharged, transferred or died on the first day are excluded.	90 %
physiotherapy	**Early physio- /occupational therapy for patients with motor disability**<br>Numerator: Patients that received treatment by physiotherapy or occupational therapy within the first two days.<br>Denominator: All patients with a paresis and clear functional deficit (modified Rankin Scale (mRS) ≥ 3 or Barthel Index ≤ 70 within the first 24 h after admission). Patients with TIA, those unconscious and those that were discharged, transferred or died on the first day are excluded.	90 %
mobilisation	**Early mobilisation of patients with severe disability**<br>Numerator: Patients that were mobilized within the first two days after admission.<br>Denominator: Patients that needed help with transfer from bed to chair (Barthel Index 0-10). Patients that were discharged, transferred or died on the first day, those with TIA and those unconscious, with mechanical ventilation or with increased intracranial pressure on admission are excluded.	90 %
atrial fibrillation screening	**Patients receiving an atrial fibrillation screening during their stay**<br>Numerator: Patients with an atrial fibrillation<br>Denominator: All patients with IS or TIA. Patients with known atrial fibrillation are excluded.	80 %

Additional file 1: Definitions of the German quality indicators in acute stroke care as defined by the Quality Indicator Board of the German Stroke Registers Study Group.

	Brief description	target value (2016)
vascular imaging	**Extracranial carotid artery diagnostic in patients with IS or TIA**<br>Numerator: Patients with imaging of extracranial vessels (doppler sonography / duplex sonography / DSA / CT-angiography / MR-angiography).<br>Denominator: All patients with TIA or cerebral infarction.	80 %
revascularisation	**Revascularisation of symptomatic carotid stenosis in patients with IS or TIA**<br>Numerator: Patients where revascularization of carotid artery was initiated or recommended.<br>Denominator: Patients with TIA or cerebral infarction with ipsilateral stenosis of carotid artery (70% - 99% NASCET) and with mRS 0-3. Patients with a mechanical recanalization are excluded.	60 %
antihypertensive drugs	**Antihypertensive drugs at discharge in patients with IS or TIA**<br>Numerator: Patients with indication for antihypertensive therapy that receive antihypertensive at discharge.<br>Denominator: All patients with IS or TIA and hypertonia as co-morbidity. Patients who died are excluded.	95 %
statins	**Patients receiving statin at discharge or whom a statin was recommended**<br>Numerator: Patients that receive statins at discharge.<br>Denominator: All patients with IS or TIA. Patients who died are excluded.	80 %
platelet inhibitors at discharge	**Platelet inhibitor given at discharge in patients with IS or TIA and no anticoagulation**<br>Numerator: All patients that receive platelet inhibition at discharge/transfer.<br>Denominator: All patients with TIA or cerebral infarction. Patients on anticoagulation and those who died are excluded as well as those aged <18 and those discharged, transferred or died on the first day.	95 %
rehabilitation	**Discharge in rehabilitation clinic**<br>Numerator: Patients where post hospital rehabilitation was initiated.<br>Denominator: All patients without previous nursing care with mRS 2-5 at discharge. Patients that were transferred or died during the hospital stay are excluded.	70 %
